# 

**Published:** 1894-11-10

**Authors:** 


**Notices of Births, Deaths, and Marriages** are inserted in this column at a charge of 2s. 6d. for an announcement not exceeding four lines.

**Notice to Advertisers and Subscribers.**

All Advertisements, Orders fo' ' opies of The Hospital, and Business Communications should be addressed to **The Publisher, NOT TO THE EDITOR**, The Hospital, 428, Strand, W.C.

The Cost of Subscription, post free, is as follows:—Per week, 2½d.; per quarter, 2s. 8d.; per half-year, 5s. 3d.; per annum, 10s. 6d. Foreign:—Per Annum, 12s. 8d.; payable, in all cases, in advance.

### NOTICE TO CORRESPONDENTS.

All MS., Letters, Books for Review, and other matters intended for the Editor should be addressed The Editor, The Lodge, Porchester Square, London, W.

The Editor cannot undertake to return rejected MS., even when accompanied by stamped directed envelope.

**"Burdett's Hospital Annual," 1894.**

Fifth year of publication. 900 pp. Boards, gilt lettering. A most complete guide to British, Colonial, Indian, and American Hospitals, Dispensaries, Nursing and Convalescent Institutions, and Asylums. Price 5s. Published by The Scientific Press (Limited), 428, Strand, W.C.

**NOTICE.—The Index for Vol. XVI. (ending September 24th, 1894) is on sale at the Office of this Journal, Price 2½d., post free.**

### The Student of Medicine.

### APPOINTMENTS.

J. Barr, M.B., J.P., reappointed Medical Officer of Health for the Rishton Urban Sanitary District. H. M. Body, M.R.C.S.Eng., L.S.A., reappointed Medical Officer of Health to the Crediton Rural Sanitary Authority. C. J. Cooke, M.D.R.U.I., M.Ch., appointed Medical Officer to the Plymouth Workhouse. J. K. Couch, M.R.C.S., L.R.C.P., late Chloroformist and Pathologist, appointed Assistant Surgeon to the Swansea Hospital. A. Gooding, L.R.C.P.Lond., M.R.C.S.Eng., appointed Surgeon to the Mission for Deep Sea Fishermen. J. T. Hartill, L.R.C.P.Lond., M.R.C.S.Eng., appointed Medical Officer of Health to the Willenhall Local Board. G. Hern, M.R.C.S., L.R.C.P., L.D.S.Eng., appointed Assistant Dental Surgeon to the Dental Hospital of London. G. J. Johnston, M.A., M.B., appointed Demonstrator of Anatomy, Trinity College, Dublin. T. Myles, M.D.Dub., appointed Medical Officer for the Bowness District of the Wigton Union. W. T. Nicholson, M.B., C.M., appointed Superintendent of the Victoria Infirmary, Glasgow. J. Shives, M.D., appointed Consulting Surgeon to the Dewsbury and District Infirmary. C. T. Urquhart, M.B.Aberd., appointed Medical Officer to the Old Mill Reformatory School, Aberdeen. F. J. Webb, M.B.Vict., Ch.B., appointed Medical Officer for the Gorton District of the Chorlton Union. W. H. Wilson, appointed House Surgeon to the St. George's Hospital. R. H. Wellington, L.R.C.P., M.R.C.S., reappointed Medical Officer of Health to the Sutton Bridge Urban Sanitary Authority, also Medical Officer to the Port of Wisbech and Port Hospital.

### VACANCIES.

### Resident Medical Officers.

Addenbrooke's Hospital, Cambridge.—Resident House Surgeon; must be duly registered. Salary £60 per annum, with board, lodging, and washing. Applications and testimonials to J. Bonnett, Secretary, 23. St. Andrew's Street, Cambridge, before November 13th.

Hospital for Sick Children, Great Ormond Street, Bloomsbury, W.C.—Resident Medical Officer as House Physician. Appointment for six months. Salary £20 per annum, with board and residence; must be unmarried. Applications and testimonials to Adrian Hope, Secretary, before November 21st.

Hospital for Sick Children, Great Ormond Street, Bloomsbury, W.C.—Resident Medical Officer as House Surgeon. Appointment for six months. Salary £20 per annum, with board and residence; must be unmarried. Applications and testimonials to Adrian Hope, Secretary, before November 21st.

Hospital for Women and Lying-in Institute, Brighton.—House Surgeon; unmarried, and under 30 years of age. Salary £80 per annum, with board and rooms. Applications to the Secretary by November 15th.

Kent and Canterbury Hospital.—House Surgeon; must be a registered medical practitioner, unmarried. Salary £90 the first year, with board, &c., rising to £100 the second year. Applications and testimonials to A. J. Lancaster, Secretary, before November 23rd.

Ratcliffe Infirmary, Oxford.—House Physician and House Surgeon. Salary £80 per annum, with board, lodging, and washing. Tenable for six months. Applications and testimonials to A. C. Virgo, Secretary, by November 16th.

### Non-Resident Medical Officers.

North-Eastern Hospital for Children, Hackney Road, Shoreditch, N.E.—Physician; must be F. or M.R.C.P.Lond. Applications to T. Glenton-Kerr, Secretary, 27, Clement's Lane, E.C., by November 15th.

Royal Infirmary, Newcastle-on-Tyne.—Two Honorary Assistant Surgeons; registered graduate in surgery, any University recognised by General Council, Medical Education, and registration of United Kingdom, or Fellow, Member, or Licentiate of one of the Royal College of Surgeons, United Kingdom. Applications to W. Oliver, Secretary, by November 7th.

**THE STUDENT OF MEDICINE**—*continued.*

MEDICAL EDUCATION IN PARIS AND GERMANY.

A Student writes : Could you tell me, through The Hospital, where I could obtain complete information about medical education in Paris and at the German universities ?

*** In the Educational Number of the *British Medical Journal* details are given of the new law regulating the practice of medicine in France, describing the various exemptions in the examinations to those who already possess qualifications, and in the Educational and Literary Supplement to the last volume of the *Medical Magazine* there will be found lists of the medical faculties in many foreign universities.—[Ed. T. H.]

### Medical Appointments.

**THE HOSPITAL FOR SICK CHILDREN,**
GREAT ORMOND STREET, BLOOMSBURY, W.C.

A. J. LANCASTER, Secretary.

Nov. 1st, 1894. (768)

**THE HOSPITAL FOR SICK CHILDREN,**
GREAT ORMOND STREET, BLOOMSBURY, W.C.

ADRIAN HOPE, Secretary.

24th October, 1894. (580)

Foolscap 8vo., cloth gilt, price 2s. (post free).

**MYXŒDEMA:**

**And the Effect of Climate on the Disease.**

By A. MARIUS WILSON, M.D., B.S., L.R.C.P.Lond., M.R.C.S.Eng.

A valuable little treatise on a subject about which comparatively little has been written. The special feature of this work is its treatment of the effects of climate upon the disease.

London: The Scientific Press (Limited), 428, Strand, W.C.

### THE SCIENTIFIC PRESS LIST.

**MEDICAL HISTORY FROM THE EARLIEST TIMES.** By E. T. WITHINGTON, M.A., M.B., Demy 8vo. Two Plates. Handsome cloth gilt. Over 400 pp. 12s. 6d. nett

**THE JOHNS HOPKINS HOSPITAL REPORTS.** Complete List on Application.

**SPINAL CURVATURE AND AWKWARD DEPORTMENT:** Their Causes, and Prevention in Children. By Dr. G. MULLER. Edited by RICHARD GREENE, F.R.C.P. Many Plates. Crown 8vo. 2s. 6d.

**INFANT FEEDING BY ARTIFICIAL MEANS.** By G. H. SADLER. Crown 8vo. Profusely illustrated. In the press.

**HOSPITALS AND ASYLUMS OF THE WORLD.** By HENRY C. BURDETT. In Four Volumes and a Portfolio of Plans. £8 8s.

**NURSING.** By ISABEL ADAMS HAMPTON. Crown 8vo. Cloth gilt. 500 pp. Plates and Illustrations. 7s. 6d. nett.

**THE MOTHER'S HELP AND GUIDE TO THE DOMESTIC MANAGEMENT OF HER CHILDREN.** By P. MURRAY BRAIDWOOD, M.D. Crown 8vo. 200 pp. Cloth, price 2s. 6d.

**HOSPITALS, DISPENSARIES AND NURSING.** Royal 8vo. 500 pages. Sixty Illustrations. Cloth gilt. Price 2s. nett.

**THE ORGANIZATION OF CHARITIES.** Cloth gilt. 8vo. 6s. nett.

**A PRACTICAL HANDBOOK OF MIDWIFERY.** By FRANCIS W. N. HAULTAIN, M.D. 18mo. Roan, Gilt Top. About 200 pages. Illustrated. 6s.

**OPHTHALMIC NURSING.** By SYDNEY STEPHENSON, M.B., F.R.C.S.E. Crown 8vo. Illustrated. Price 3s. 6d.

**BURDETT'S HOSPITAL AND CHARITIES ANNUAL 1894.** By HENRY C. BURDETT. 5s.

**ACCOUNTS, AUDIT, AND TENDERS FOR HOSPITALS AND INSTITUTIONS,** the Uniform System of. By HENRY C. BURDETT. 6s.

**THE THEORY AND PRACTICE OF NURSING.** By PERCY G. LEWIS, M.D. 7th Edition. 3s. 6d.

**MENTAL NURSING.** By WILLIAM HARDING, M.B. Second and Revised Edition, with additional chapters. 2s. 6d.

**HOW TO BECOME A NURSE: And How to Succeed.** By HONNOR MORTEN Profusely illustrated. 2s. 6d.

**ART OF FEEDING THE INVALID.** By a MEDICAL PRACTITIONER and a LADY PROFESSOR OF COOKERY. 3s. 6d.

**ART OF MASSAGE.** By A. CREIGHTON HALE. Seventy Illustrations. 6s.

**SURGICAL WARD-WORK AND NURSING.** By ALEXANDER MILES, M.B. (Edin.), C.M., F.R.C.S.E. Demy 8vo. Many Illustrations. Cloth extra. 3s. 6d.

**OUTLINES OF INSANITY.** By FRANCIS H. WALMSLEY, M.D. Cloth, 3s. 6d. Popular Edition, 1s. 6d.

**HOSPITAL SISTERS AND THEIR DUTIES.** By EVA C. E. LUCKES. 2s. 6d.

**THE NURSES' DICTIONARY OF MEDICAL TERMS AND NURSING TREATMENT.** By HONNOR MORTEN. Second and Revised Edition. 15th Thousand. 2s.

**MINISTERING WOMEN: THE STORY OF THE ROYAL NATIONAL PENSION FUND FOR NURSES.** By GEORGE W. POTTER, M.D. 2s. 6d.

London: THE SCIENTIFIC PRESS (Limited), 428, Strand, W.C.

